# The Proteomic and Peptidomic Response of Wheat (*Triticum aestivum* L.) to Drought Stress

**DOI:** 10.3390/plants14142168

**Published:** 2025-07-14

**Authors:** Regina Azarkina, Arina Makeeva, Anna Mamaeva, Sergey Kovalchuk, Daria Ganaeva, Igor Tikhonovich, Igor Fesenko

**Affiliations:** 1 Shemyakin-Ovchinnikov Institute of Bioorganic Chemistry of the Russian Academy of Sciences, Moscow 117997, Russia; aryamakeeva@gmail.com (A.M.); physcomitrella.proteomics@gmail.com (A.M.); xerx222@gmail.com (S.K.);; 2 Laboratory of System Analysis of Proteins and Peptides, Shemyakin and Ovchinnikov Institute of Bioorganic Chemistry, Russian Academy of Sciences, Moscow 117997, Russia; 3 All-Russia Research Institute for Agricultural Microbiology, Podbelscogo hwy 3, Pushkin, St. Petersburg 196608, Russia; 4 Department of Genetics and Biotechnology, Faculty of Biology, Saint Petersburg State University, 7-9 Universitetskaya Embankment, Saint Petersburg 199034, Russia; 5 Independent Researcher, Moscow 117997, Russia; fesigor@gmail.com

**Keywords:** proteome, peptidome, secretome, drought, LC-MS/MS, iTRAQ, wheat

## Abstract

Drought conditions impact plants at the morphological, physiological, and molecular levels. Plant tolerance to drought conditions is frequently associated with maintaining proteome stability, highlighting the significance of proteomic analysis in understanding the mechanisms underlying plant resilience. Here, we performed proteomic and peptidomic analysis of spring wheat (*Triticum aestivum* L.) under drought stress conditions. Using isobaric tags for relative and absolute quantitation (iTRAQ), we identified 497 and 157 differentially abundant protein (DAP) groups in leaves and roots, respectively. The upregulated DAP groups in leaves were primarily involved in stress responses, such as oxidative stress and heat response, whereas those in roots were associated with responses to water deprivation and sulfur compound metabolic processes. The analysis of the extracellular root peptidome revealed 2294 native peptides, including members of small secreted peptide (SSP) families. In the peptidomes of stress-induced plants, we identified 16 SSPs as well as peptides derived from proteins involved in cell wall catabolism, intercellular signaling, and stress response. These peptides represent potential candidates as regulators of drought responses. Our results help us to understand adaptation mechanisms and develop new agricultural technologies to increase productivity.

## 1. Introduction

Drought is one of the major factors contributing to global yield losses, accounting for up to 50% of crop reductions worldwide [1]. The impact of drought is particularly severe during specific developmental stages, such as fruit formation, often resulting in substantial yield losses. Historically, severe drought conditions have repeatedly resulted in widespread famine and continue to pose a significant threat to global food security and agricultural sustainability [2,3].

Drought affects plant tissues and organs by membrane disintegration, protein degradation, and growth suppression, primarily due to inhibited cell elongation [4]. To maintain cellular osmotic balance and counteract turgor loss, plants accumulate osmoprotectants, such as proline, glycine, betaine, and various sugars [5]. These molecules not only stabilize proteins and membranes but also scavenge reactive oxygen species (ROS). Indeed, drought-induced oxidative stress results from excessive ROS production [6]. In response, plants upregulate antioxidant defense systems, including enzymatic components such as superoxide dismutase (SOD), catalase (CAT), and various peroxidases (POD) [7]. Plants also induce the expression of Late Embryogenesis Abundant (LEA) proteins and dehydrins (DHNs), which function as molecular chaperones, protecting macromolecules and cellular structures from damage by stabilizing proteins and membranes [4,8].

Drought-tolerant cultivars elevated levels of LEA proteins, DHNs, heat shock proteins, and enzymes linked to antioxidant defense and osmotic adjustment under water deficit [9]. These changes are the result of both transcriptional reprogramming and selective accumulation and stabilization of proteins that safeguard cellular structures and metabolic homeostasis [9,10].

Besides proteins, Small Secreted Peptides (SSPs) were shown to play an important role as key signaling molecules under drought stress [11,12,13,14,15,16,17]. SSPs are derived from protein precursors that contain a signal peptide. They have been shown to play a role as mediators of intercellular communication [18,19,20]. Exogenous application of SSPs from specific families has been shown to enhance drought tolerance. For example, treatment with synthetic CLAVATA3 (CLV3)/Embryo Surrounding Region 25 (CLE25), C-terminally Encoded Peptide 5 (CEP5), and overexpression of peptide hormone *CLAVATA3/ESR-RELATED 9* (*CLE9*) in *Arabidopsis thaliana* increases drought tolerance [21,22,23]. Also, the drought-induced peptide Rapid Alkalinization Factor 8 (RALF8) in *A. thaliana* facilitates cell wall remodeling under water deficit [24]. The premature drought-induced leaf drop is shown to be regulated by phytosulfokine (PSK) and Inflorescence Deficient in Abscission (IDA) peptides in tomatoes and *A. thaliana*, respectively [25,26]. Also, some members of the Epidermal Patterning Factor-Like (EPFL) peptide family modulate drought response [27,28,29].

In addition to SSPs, peptides, which are released from functional proteins, can be involved in the plant stress response [13,30,31,32,33,34]. In contrast to animals, the functional roles of such peptides are mostly unknown [35]. However, isolation and identification of both SSPs and degradome-derived peptides are difficult due to their very low abundance in vivo [36].

Wheat is one of the most important cereal crops in the world [37]. The development of proteomic, genomic, and transcriptomic technologies led to the identification of new drought-responsive wheat proteins that have potential for use in marker-assisted selection [38]. In particular, analysis of the developing grains’ transcriptome revealed a large number of drought-responsive genes associated with water regulation, osmotic stress, and stress signaling [39]. The transcriptome analysis of wheat roots under drought conditions revealed significant upregulation of genes encoding DHNs, aquaporins, and various transcriptional factors [40,41]. In addition, 15 calmodulin (*TaCAM*) and 113 calmodulin-like (*TaCML*) genes were upregulated under drought and salt stresses [42]. Long-term water deprivation leads to transcriptional changes of thousands of genes, most of which belong to flavonoid biosynthesis, plant hormone signaling, and the antioxidant pathway in wheat [43].

Numerous proteomic studies have investigated wheat responses to drought stress [44,45,46,47,48,49,50,51,52]. Most of these focused on individual organs such as leaves, grains, or roots. These studies identified drought-responsive proteins involved in various metabolic pathways, including sugar and amino acid synthesis, antioxidant defense, cytoskeletal stabilization, and signal transduction [45,46,47,48,49,50,51,52]. For example, proteins involved in stress defense and detoxification, such as heat shock proteins (HSPs) and ROS scavenging proteins (ascorbate peroxidase, glutathione S-transferase (GST), thiol-specific antioxidant protein), are upregulated under drought conditions in wheat [49,53]. In addition, chaperones and enzymes involved in proline biosynthesis also show increased abundance. Furthermore, proteins associated with cell biogenesis and degradation are upregulated in the leaves of stress-tolerant wheat genotypes [54].

The studies on wheat grains have focused on developmental and storage processes affected by water deficit [46]. It has been demonstrated that albumin and gliadin are significantly upregulated under drought stress [46]. Proteomic analysis of wheat seed germination under drought stress conditions revealed significant effects of pathways involved in phenylpropanoid metabolism and fatty acid biosynthesis [55]. Although the wheat root proteome response to drought conditions has been less extensively studied, available data highlight the importance of proteins involved in water uptake, carbon metabolism, and nutrient metabolism [47,49]. The first comprehensive root proteome study showed that HSPs, O-methyltransferase, and 2-caffeoyl CoA-methyltransferase were upregulated in tolerant genotypes [56]. Despite extensive research on proteins, the role of bioactive peptides, such as SSPs, remains relatively understudied [57].

In this study, we performed a quantitative analysis of the leaf and root proteomes as well as the extracellular root peptidomes of spring wheat under osmotic stress. Our study provided a better understanding of the drought adaptation mechanisms in wheat and the potential development of technologies to improve drought tolerance.

## 2. Results

### 2.1. Morphological Changes in Wheat Plants Under Drought Stress

To analyze proteomic and peptidomic changes under drought conditions in wheat, we selected the commonly used early-maturing spring wheat cultivar Leningradskaya 6, which is adapted to the growing conditions in the northwest regions of Russia [58] and has shown the strongest response to inoculation with plant growth-promoting *Bacillus* sp. *V2026* [59]. In plants, drought stress can be induced through various methods [60]. Due to our interest in the analysis of the extracellular wheat peptidome, wheat plants were grown in a hydroponic system, and the polyethylene glycol (PEG-6000) was used as an osmotic stressor [49,61]. To simulate drought stress, wheat plants were incubated with 20% PEG-6000 for 4 and 8 days in the hydroponic system.

**Figure 1:**
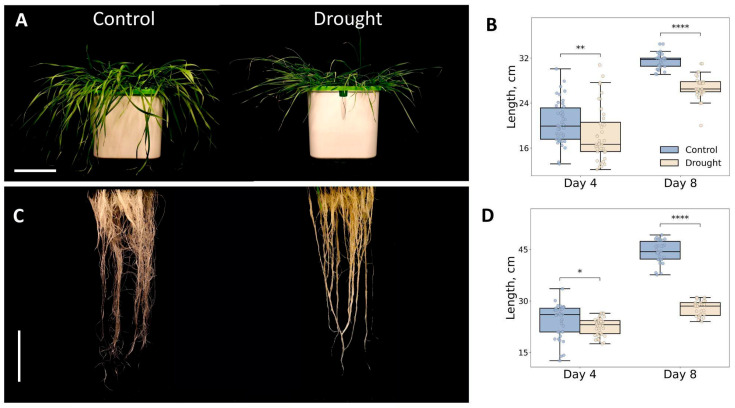
Morphological changes in seedlings of wheat (*Triticum aestivum* L., Leningradskaya 6 cultivar) under 8-day treatment with PEG 20%. (**A**) Shoots of plants under 8-day treatment with PEG 20% and control plants. Scale bar = 10 cm. (**B**) Changes in leaf length after 4 and 8 days of treatment with 20% PEG. Error bars indicate the standard error of three biological replicates. (**C**) Roots of plants under 8-day treatment with PEG 20% and control plants. Scale bar = 10 cm. (**D**) Changes in root length under 4 and 8 days treatment with PEG-20%. Error bars indicate the standard error of three biological replicates. Statistical significance was assessed using a two-tailed Mann–Whitney U test. Asterisks indicate significance levels: * *p* < 0.05, ** *p* < 0.01, **** *p* < 0.0001.

The most pronounced reduction in root and leaf length was observed on day 8 (Figure 1); therefore, further analyses were conducted on plants incubated under drought conditions for 8 days.

### 2.2. Drought Conditions Result in Significant Proteome Changes

**Figure 2:**
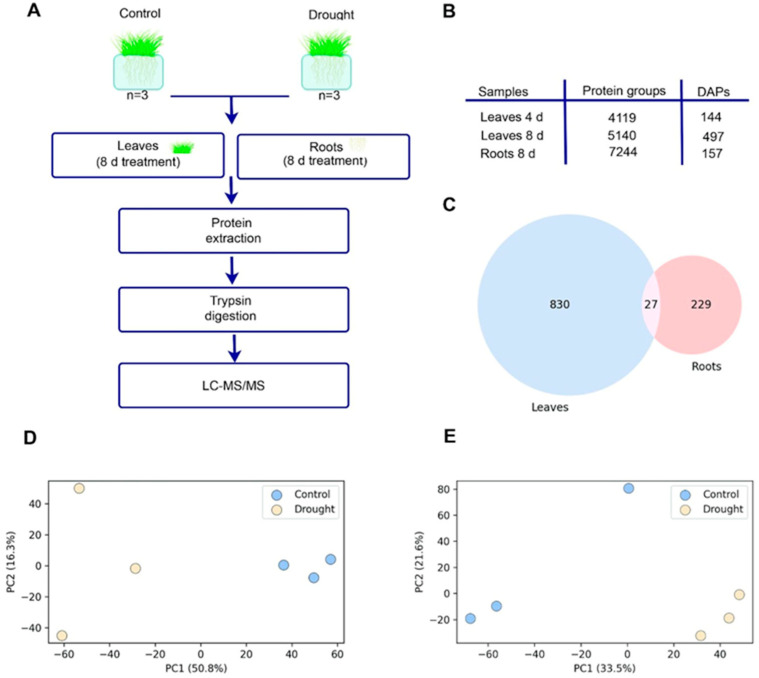
Analysis of the proteome. (**A**) Overview of the experimental workflow. (**B**) Quantitative proteome analysis of wheat under 8-day drought stress. (**C**) Venn diagram showing the overlap of DAPs between the leaves and roots’ samples. The intersection represents proteins commonly regulated in both tissues. (**D**) PCA of wheat under drought for 8 days and controls in leaves. (**E**) PCA of wheat under drought for 8 days and controls in the roots.

At first, quantitative proteomic analysis using iTRAQ labeling was performed on leaves collected after 4 and 8 days of treatment with 20% PEG-6000. We identified 41 differentially abundant proteins (DAPs) concordantly changed across these time points, further supporting the reproducibility of our analysis (Tables S1–S3). Since the most pronounced changes in protein abundance were observed on day 8, further analysis focused on plants incubated under drought conditions for 8 days (Figure 2B, Tables S1 and S2).

In wheat leaves, we identified 857 DAPs, which were assigned to 497 (239 up- and 258 down-regulated) DAP groups (Figure 2B, Table S2) under 8 days of drought stress. The proteomic changes in roots were less pronounced and included 256 DAPs, which were assigned to 157 (102 up- and 55 down-regulated) DAP groups (Figure 2B; Table S4) under 8 days of drought stress. Since a protein group may contain multiple putative proteins, we performed a comparative analysis between all DAPs in roots and leaves. However, only 27 DAPs were shared between roots and leaves, indicating minimal overlap (Figure 2C). Among these, 4 proteins were down-regulated in both organs, 20—upregulated, and 2 proteins showed opposite regulation patterns between leaves and roots (Table S5).

PCA demonstrated a high degree of consistency among the biological replicates in two groups (Figure 2D,E), indicating that drought stress caused a significant difference in protein expression.

**Figure 3:**
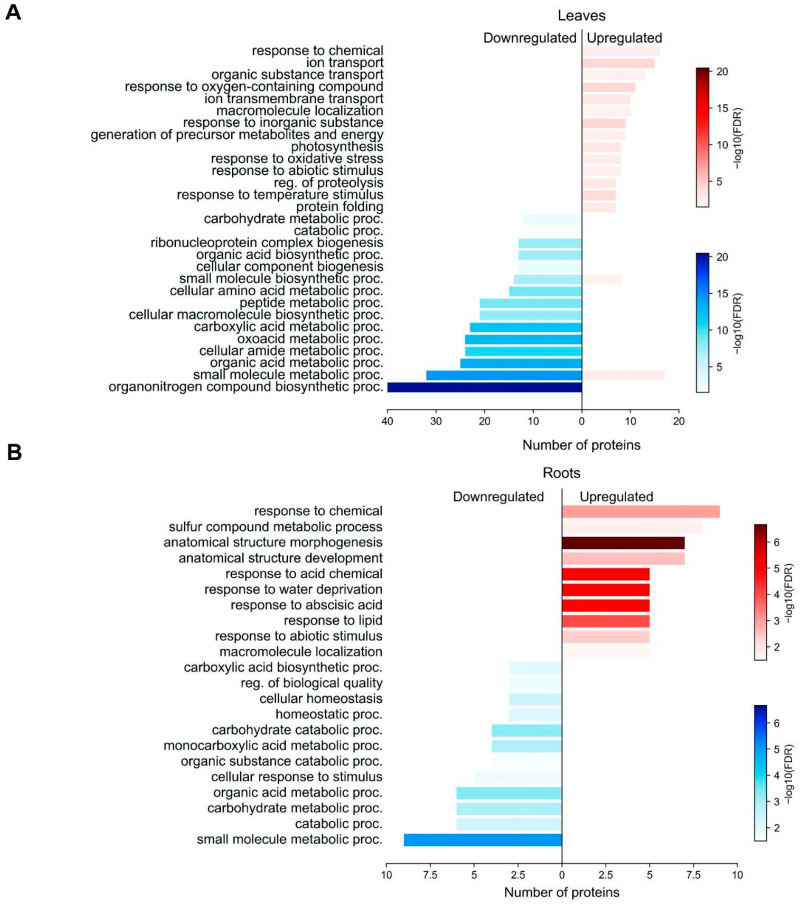
The GO term analysis of wheat. (**A**) GO terms of DAP groups involved in biological processes in leaves after the 8 d drought. (**B**) GO terms of DAP groups involved in biological processes in roots after the 8 d drought. The X-axis represents the number of proteins in each GO Term. The Y-axis is GO Terms. Enrichment significance is indicated by –log_10_(FDR) values displayed next to each bar. GO analysis was carried out by ShinyGO 0.80.

**Figure 4:**
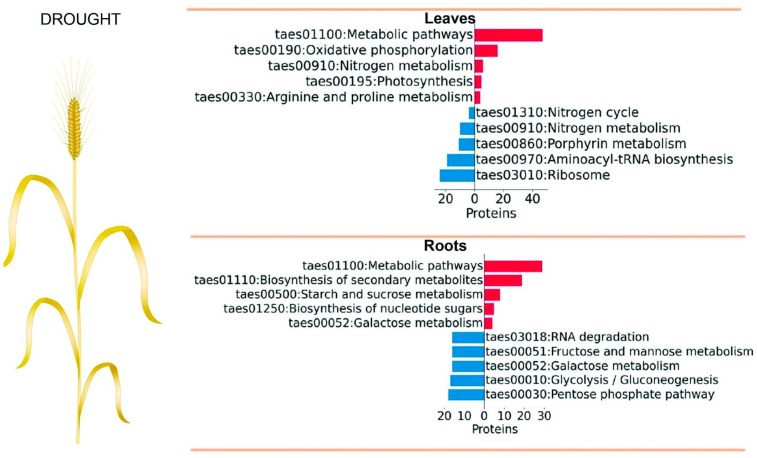
The KEGG annotation for the DAP groups under drought stress. Red bars indicate KEGG pathways enriched among upregulated DAP groups, while blue bars represent those enriched among down-regulated DAP groups. The number of associated proteins is shown along the x-axis. Enrichment significance is indicated by −log_10_(FDR) values displayed next to each bar.

The GO term analysis provides insights into the molecular pathways that enable plants to adapt to environmental stresses. We next performed GO term annotation of DAP groups using the bioinformatic tool ShinyGO 0.80 [62]. Among the 497 DAP groups, 339 (69%) were successfully annotated at the GO level in leaves and 99 (63%) of the 157 DAP groups in roots. We found that upregulated DAP groups in leaves were enriched in such GO terms as the responses to different stimuli and stress conditions, response to oxygen-containing compounds (GO:1901700), obsolete response to inorganic substances (GO:0010035), and response to temperature stimulus (GO:0009266; Figure 3). The upregulated DAP groups in leaves were enriched in such KEGG pathways as oxidative phosphorylation and nitrogen metabolism (Figure 4). The down-regulated DAP groups were enriched in such GO terms as catabolic process (GO:0009056), cellular macromolecule biosynthetic process (GO:0034645), and peptide metabolic process (GO:0006518) in Figure 3, indicating a decline in cytoskeletal dynamics under drought.

**Figure 5:**
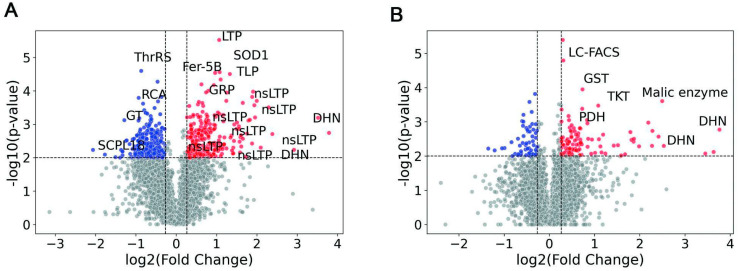
(**A**) Volcano plot indicating DAP groups present in leaves. (**B**) Volcano plot indicating DAP groups present in roots. Each spot represents one protein. On the y-axis, −Log_10_ (*p*-value) is indicated. On the x-axis, Log_2_ (fold-change) is shown; negative values indicate proteins that were down-regulated under drought conditions, while positive values indicate proteins that were upregulated. Blue dots correspond to proteins that were significantly down-regulated, red dots represent significantly upregulated proteins, and grey dots represent non-regulated proteins.

In leaves, the most strongly upregulated proteins belonged to well-known stress-responsive groups, including DHNs, RNA-recognition motif (RRM) domain-containing proteins, non-specific lipid-transfer proteins (nsLTPs), and water stress and hypersensitive response domain-containing proteins (Figure 5A; Table S2). We also identified seven significantly upregulated nsLTPs (Figure 5A) exclusively in leaves but not in roots. The identification of DAP groups associated with lignin biosynthesis (dirigent protein (DIR)) and stress-related functions (nsLTPs) shows that plants employ a multifaceted strategy to manage drought stress.

The upregulated DAP groups in roots were enriched in such GO terms as response to chemical stimulus (GO:0042221), water deprivation (GO:0009414), and sulfur compound metabolic process (GO:0006790) (Figure 3). The most enriched KEGG pathways that involved upregulated proteins were starch and sucrose metabolism, biosynthesis of secondary metabolites, biosynthesis of nucleotide sugars, and galactose metabolism (Figure 4). The changes in the starch and sucrose metabolism pathways indicate that the plants increase sugar biosynthesis in response to drought stress.

Root proteome analysis revealed pronounced changes in proteins involved in stress adaptation and redox regulation under drought conditions. Upregulated proteins included DHNs, which are well-established protective molecules stabilizing cellular structures during dehydration (Figure 5B; Table S4). Notably, malic enzyme (ME), an important player in carbon metabolism and NADPH production, was markedly increased. LEA proteins and SMP domain-containing proteins, associated with osmoprotection and membrane stabilization, were also significantly enriched, underscoring multifaceted protective strategies employed by wheat roots in drought (Figure 2E; Table S4).

Several proteins were consistently upregulated under drought conditions in both leaves and roots. These included multiple dehydrins (A0A3B6QLW3, A0A3B6LSK1, A0A3B6LUF1), a salt-induced YSK2 dehydrin (W5GW81), a LEA protein (A0A024CKY0), an S-like RNase (Q6R326), and an uncharacterized protein (A0A3B6QLV9) (Figure 2C, Table S5). Additionally, EF-hand and PH domain-containing proteins were commonly regulated across both tissues, pointing to potential roles in calcium signaling and membrane-associated stress responses. This overlap highlights a conserved core of drought-responsive proteins in wheat, irrespective of tissue type.

A small set of proteins was consistently down-regulated in both roots and leaves under drought conditions. These included a 14-3-3 protein (G5DFC5), tripeptidyl-peptidase II (A0A3B6NRW7), lysine–tRNA ligase (A0A3B6MZJ5), and a LURP-one-related protein (A0A1D6B0F9). Their consistent repression suggests that drought stress may suppress specific components of protein synthesis, proteolysis, and signaling, potentially as part of an energy-saving or growth-inhibitory response.

### 2.3. Extracellular Peptides in Root Secretome Under Drought Conditions

**Figure 6:**
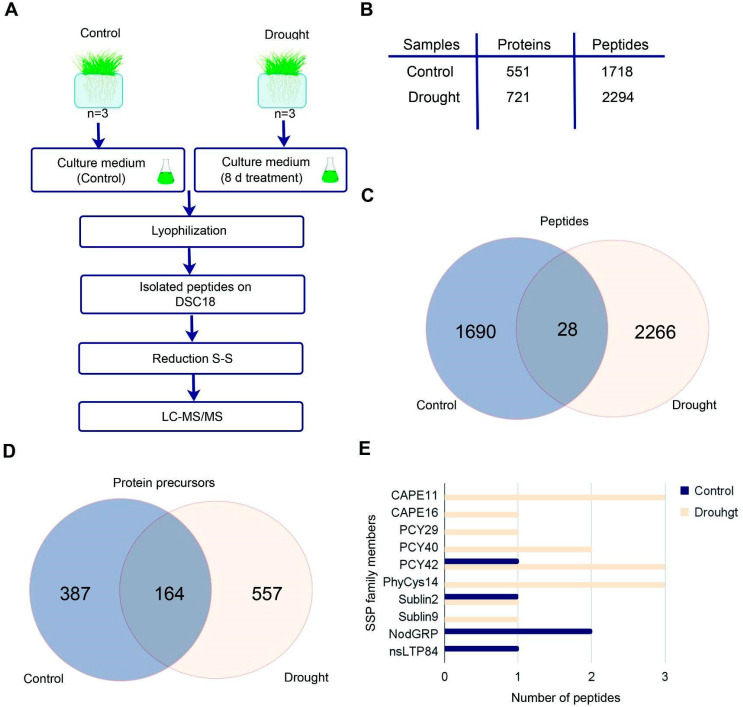
Analysis of secretome peptides. (**A**) Overview of the experimental workflow. (**B**) Characteristics of the peptidome dataset after PEG treatment for 8 days. (**C**) Venn diagram showing comparison of identified endogenous peptides between control and PEG 20% treatment. (**D**) Venn diagram showing comparison of identified protein precursors of peptides between control and PEG 20% treatment. (**E**) The families of SSP are identified in the extracellular peptidome.

Peptides are key signaling molecules in the communication between roots and leaves, thereby activating plant defenses and physiological changes that promote tolerance to stress [22,25,63,64]. We suggested that the stress peptidome, which includes cryptic bioactive peptides released during the degradation of functionally active proteins [35], might be a part of the plant’s drought response. It has been shown previously that secreted peptides from roots would diffuse in the culture medium [65]. Therefore, to identify biologically active secreted peptides, wheat plants were grown in a hydroponic system under normal and drought conditions for 8 days (Figure 6A), and native peptides were extracted from the culture medium. Using top-down mass spectrometry analysis, we identified 1718 peptides derived from 551 protein precursors in normal conditions (Figure 6B; Table S6). In addition, we identified 2294 peptides derived from 721 protein precursors under PEG treatment (Figure 6B,C; Table S7). The GO term analysis showed that the majority of the protein precursors participate in cell wall metabolism, peptide metabolism, and purine ribonucleotide metabolism (Figure S1).

SSPs are known to play a critical role in intercellular communication and drought stress response [66,67]. Therefore, we additionally searched for evidence of SSPs translation in our peptidomic data. For this analysis, protein precursor sequences of identified peptides (Tables S6 and S7) were re-annotated using the Small Secreted Peptide Database [68]. Under normal conditions, we obtained the evidence of translation for 4 SSPs from four families—Plantacyanin (PCY), nsLTP, Subtilisin inhibitor (SubIn), and Nodule-specific Glycine-rich Protein (NodGRP) (Table S8). In drought stress, we obtained the evidence of translation for 16 SSPs from 4 SSP families, including phytocystatins (PhyCys), PCY, CAP-derived peptide (CAPE), and SubIn (Figure 6E; Table S8). Furthermore, we detected an increased number of Plantacyanin/Chemocyanin (PCY) families under drought stress. Additionally, our proteomic data revealed the presence of PhyCys and PCY families under drought stress (Table S8).

Following the identification of SSPs in the extracellular peptidome, we re-analyzed publicly available RNA-seq data on drought-stressed wheat roots of two *T. aestivum* cultivars—ZM366 and CM42 [41] to assess the expression patterns of SSP-encoding genes. Our analysis identified 22 transcripts from 9 SSP families that were upregulated under drought conditions (log_2_FC > 1, adjusted *p*-value < 0.05) and 14 down-regulated transcripts from 6 SSP families (log_2_FC < −1, adjusted *p*-value < 0.05) in the CM42 cultivar (Table S9). In the ZM366 cultivar, drought stress resulted in upregulation of 39 transcripts from 14 SSP families and down-regulation of 12 transcripts from 7 SSP families (Table S10).

In both lines, there were significant changes in the transcription of cysteine-rich SSP genes, including *PCY*, *CAP-derived peptide* (*CAPE*), *nsLTP*, *Hevein*, and *Bougainvillea bract protein inhibitor* (*BBPI*). Thus, we observed a notable increase in the transcriptional level of cysteine-rich SSP families, particularly PCY, that were also identified in extracellular peptidomes (Figure 6E; Table S9). We also observed an upregulation of protease inhibitor PhyCys at both transcriptome, proteome, and peptidome levels. Additionally, the ZM366 line showed a significantly increased expression of 17 members of the nsLTP family, which is known to facilitate lipid transport. Therefore, we detected changes in the abundance of nsLTPs across both the transcriptomic and proteomic datasets, which indicates their relevance in the plant drought stress response. The difference in SSPs’ expression at the transcriptomic level between the two cultivars suggests potential molecular mechanisms that enable different wheat lines to withstand such stresses.

## 3. Discussion

In this study, we performed proteomic and peptidomic analysis of the early-maturing spring wheat cultivar Leningradskaya 6 under drought stress conditions. Our analysis revealed DAP groups involved in stress responses, such as oxidative stress and heat response in leaves, and associated with responses to water deprivation and sulfur compound metabolic processes in roots. Notably, only a small subset of proteins was shared between the two organs, reflecting distinct tissue-specific drought adaptation strategies.

Several proteomic studies have previously addressed drought responses in wheat, but these typically focused on individual organs and early stress stages. For example, Ford et al. (2011) conducted a shotgun proteomic analysis of leaves and identified 159 DAP groups related to photosynthesis and ROS scavenging [45]. Zhang et al. (2014) compared leaf and grain tissues, highlighting tissue-specific responses, but did not examine roots [46].

In contrast to previous studies, we utilized iTRAQ-based quantitative proteomic analysis to investigate the late-phase adaptive responses in both leaf and root proteomes, which had not been described previously. The depth of our dataset also allowed us to perform a more detailed comparison of proteomic changes between different organs [69]. Consistent with earlier findings [51], we observed clear differences in drought responses between roots and leaves. Notably, only a small subset of proteins was shared between the two organs, highlighting distinct tissue-specific adaptation strategies to drought stress. Furthermore, the overall magnitude of proteomic changes was smaller in roots compared to leaves.

Consistent with prior research, we identified DHNs as the most upregulated proteins in both leaves and roots, confirming their role in enhancing drought tolerance by stabilizing proteins and membranes during desiccation [70]. Similarly, the upregulation of SODs, GSTs and peroxidases corroborates previous findings about the activation of antioxidant systems protecting cells from oxidative damage [71,72].

Previous studies have shown that overexpression of the Photosystem II reaction center Psb28 subunit impaired drought tolerance in transgenic *Arabidopsis thaliana* [73]. In our analysis, the Photosystem II reaction center Psb28 protein was significantly upregulated in wheat leaves under drought stress, suggesting its involvement in the drought response.

In leaves, we observed increased levels of DIR proteins (A0A3B6FG70; A0A341W7H8; A0A3B6SD56), which regulate lignin biosynthesis and contribute to cell wall reinforcement, as well as RRM domain-containing proteins (A0A3B6REA1, A0A3B6KQ24, A0A3B6MJX3), likely involved in post-transcriptional regulation under stress [74,75].

Our study highlights the upregulation of seven non-specific nsLTPs in leaves, which have been implicated in stress responses, symbiosis establishment, and immune reactions [76,77,78]. The fact that two of these proteins (A0A3B6GKQ2; W5D2I6) have also been identified in wheat in other studies acknowledges their importance and suggests a conserved role in the plant’s defense mechanisms against environmental stressors [79]. The tissue-specific presence of these nsLTPs, supported by increased transcript levels, suggests that they have a specific function in protecting leaf tissues from drought-induced damage. Furthermore, we detected differential regulation of calcium-binding proteins, such as calreticulin, which decreased under drought, and calcium-dependent protein kinases, some of which were upregulated, indicating a complex modulation of calcium signaling pathways [80].

Another intriguing aspect of our data is the involvement of sulfur metabolism in drought adaptation. Upregulated proteins linked to sulfur-containing compounds, such as glutathione transferase (A0A3B6GSQ6) and 5-methyltetrahydropteroyltriglutamate–homocysteine S-methyltransferase (A0A3B6MJZ2), point to enhanced antioxidant capacity and metabolic adjustments, consistent with previous reports on the importance of sulfur in stress adaptation [81,82]. Meanwhile, the down-regulation of sulfotransferase (A0A3B5Y3I8) in roots may reflect organ-specific metabolic shifts.

In addition to the proteomic analysis, we examined extracellular peptide pools under control and drought conditions, identifying over 2000 endogenous peptides, including 16 SSPs in the root secretome. Although secreted peptides are increasingly recognized as mediators of stress responses, such data for wheat under drought conditions have not been reported previously.

Intercellular communication between roots and leaves is crucial for the proper activation of plant defenses and physiological changes that promote tolerance to stress. Plantacyanins are components of the plant cell wall and play various roles in plant development and stress response [83,84]. It is shown that PCY is involved in the regulation of seed germination, anther development, and pollen tube chemotropism [84,85,86]. Moreover, drought-induced expression of *PCY* in the stomatal guard cells increases the ROS level, leading to stomatal closure and enhanced drought tolerance [83]. Our peptidomic analysis and publicly available RNA-seq data of drought-stressed wheat roots both revealed a notable upregulation of the PCY family peptides under drought stress.

We also observed the consistent presence and upregulation of PhyCys family members both in the extracellular root peptidomes and transcriptome in response to drought stress. While their classical function involves inhibiting papain-like cysteine proteases (PLCPs), several family members have been implicated in responses to abiotic stresses such as salt, drought, and heat [87,88,89]. Previous transcriptomic analyses have shown that *Oryza sativa* PhyCys14 is induced under drought conditions, yet its specific functional role remains poorly characterized [90,91].

Among the peptides identified in the extracellular root secretome under drought stress, we also detected CAPE11 and CAPE9—members of the CAPE family derived from the precursor pathogenesis-related protein 1 (PR-1). CAPEs have been previously implicated in immune signaling; for instance, CAPE1 functions as a damage-associated molecular pattern (DAMP) that activates jasmonic acid and ethylene signaling pathways to promote immune responses. Recent work has shown that CAPE1 expression is down-regulated under salt stress, and its reduced levels are associated with diminished salt tolerance [92]. In our study, however, CAPE9 and CAPE11 were consistently detected in the drought-stressed wheat root secretome, suggesting a possible role in the drought response that has not been previously described.

Taken together, these results highlight the potential significance of specific SSP families in increased plant resistance to abiotic stress, suggesting a need for further research to understand their functions and mechanisms in drought tolerance.

## 4. Materials and Methods

### 4.1. Plant Growth Conditions and Treatments

The seed of the commonly used early-maturing spring wheat cultivar Leningradskaya 6, which is adapted to the growing conditions in the northwest regions of Russia [58], has shown the strongest response to inoculation with plant growth-promoting *Bacillus* sp. *V2026* (https://doi.org/10.3390/plants11141817) was obtained from the All-Russia Research Institute of Agricultural Microbiology, St. Petersburg, Russia [58,93]. The seeds were sterilized by 0.1% sodium hypochlorite (NaClO) solution for 10 min, washed 7 times, and then germinated for 24 h on wet filter paper at room temperature in the dark. The germinated seeds were transferred to a cultivation plastic container (190 × 190 × 175 cm) with 3 L full-strength Hoagland solution [94] in growth chambers (*Panasonic* PHCBI *MLR*-*352H*-*PE* Plant Growth Humidity Light Klima Schrank Cabinet, Tokyo, Japan) with a temperature of 22 ± 2 °C under 16 h photoperiod under photosynthetically active light (250 µmole m^−2^ s^−1^). The pH (6.0–6.5) was optimized by adding NaOH. Fresh air was supplied in solution by an aeration pump (220 V). The nutritional solution was changed every 3 days. Drought stress was imposed on seedlings starting from the three-leaf stage by adding 20% *w*/*v* PEG-6000 (polyethylene glycol-6000) to the Hoagland solution to obtain an osmotic potential (ψ s) of –0.75 MPa [49]. Samples were collected after 8 days of drought treatment. Plant materials were stored at –80 °C prior to use. The experiments were conducted in three biological replicates.

### 4.2. Protein Extraction

Protein extraction from leaves and roots and following trypsin digestion were conducted as previously described [92], with three biological replicates for each treatment. Briefly, proteins were extracted using the phenol extraction method [95], dissolved in Sample buffer (8 M urea, 2 M thiourea, 10 mM Tris), quantified by Bradford protein assays (Bio-Rad), reduced in 5 mM dithiothreitol for 30 min at 50 °C, and then alkylated by 10 mM iodoacetamide for 20 min. Obtained proteins were dissolved four times in 40 mM ammonium bicarbonate and digested by incubating with trypsin (Promega) overnight at 37 °C.

### 4.3. iTRAQ Labeling and Cation Exchange Fractionation

Proteins were labeled with the iTRAQ tags as follows: leaves, control—113, 115, and 119 tags, 8 days of drought stress—114, 116, and 121 tags; roots, control—115, 117, and 119 tags, 8 days of drought stress—116, 118, and 121 tags. Samples were mixed, vacuum-dried, and dissolved in 1% trifluoroacetic acid (TFA). To increase the number of identified proteins, labeled peptides were fractionated by cation exchange chromatography on strong cation exchange (SCX) extraction disks (Supelco, Bellefonte, PA, USA). Peptides were eluted successively by 50, 75, 125, 200, and 300 mM ammonium acetate in 0.5% formic acid and 20% acetonitrile; 5% NH_4_OH in 80% acetonitrile; 10% NH_4_OH in 60% acetonitrile. This procedure has been shown not to significantly affect protein quantification by iTRAQ (Hao et al. 2015 [49]). A total of 6 fractions were collected, and each fraction was dried in a vacuum concentrator for the next step. Peptide fractions were resuspended with 100 μL solvent (water with 0.1% formic acid) and desalted on DSC-C18 extraction disk columns and were washed with 0.1% TFA and eluted with 80% acetonitrile with 0.1% TFA, and each fraction was dried in a vacuum concentrator for the next step.

### 4.4. LC-MS/MS Analysis

LC-MS analysis was carried out using an UltiMate 3000 RSLCnano HPLC system connected to an Orbitrap Fusion Lumos mass spectrometer (ThermoFisher Scientific, Markham, ON, Canada). Samples were loaded to a home-made trap column 40 × 0.1 mm, packed with Reprosil Pur C18 5 um (MZ-ANALYSENTECHNIK GmbH, Mainz, Germany) in the loading buffer (2% ACN, 98% H_2_O, 0.1% TFA) at 4.5 µL/min flow and separated at RT in a fused-silica column 300 × 0.1 mm packed with Reprosil PUR C18AQ 1.9 (MZ-ANALYSENTECHNIK GmbH, Mainz, Germany) into the emitter prepared with P2000 Laser Puller (AutoMate Scientific, San Diego, CA, USA) [96]. Fractionated samples were eluted with a linear gradient of 80% ACN, 19.9% H_2_O, 0.1% FA (buffer B) in 99.9% H_2_O, and 0.1% FA (solvent A) from 8 to 45% of solvent B in 2 h at 0.5 µL/min flow at RT. MS data were collected in DDA mode with 2 s cycle time. MS1 parameters were as follows: 120K resolution, 350–1500 scan range, injection time, and AGC target set to auto. Ions were isolated with a 1.4 m/z window, charge +2 to +7, and 5 × 10^4^ intensity threshold. Dynamic exclusion was set to 40 s. MS2 fragmentation was carried out in HCD mode at 15K resolution with stepped CE 30, 35, and 40%. The initial mass was set to 100. AGC (Automatic Gain Control) target and injection time set to auto.

### 4.5. Extracellular Peptides Extraction

For the peptide assays, wheat plants were grown in a hydroponic system under normal and drought conditions for 8 days. Then the wheat seedlings were cultured with Hoagland nutrient solution without PEG-6000 for an additional day. The Hoagland solution was sterilized by filtration through a 0.22 μm membrane filter (Millipore, Burlington, MA, USA). This incubation aimed to establish a controlled environment for the effective extraction of extracellular peptides. Following this, 400 mL of wheat culture medium was collected for peptides’ extraction. The culture medium was filtered through a 0.22 μm membrane filter (Millipore), lyophilized, and isolated on reversed-phase SPE (Solid-Phase Extraction) cartridges DSC-18 (Discovery DSC-18, Supelco, USA) using 500 μL 50% acetonitrile with 0.1% TFA solutions for the elution. The eluted peptides were concentrated in a SpeedVac to 5 μL and followed by the reduction of disulfide bonds as previously described [97]. For this peptide, samples were resuspended in 40 μL disulfide reduction solution (0.02 M CAA, 0.025 M TCEP, and 0.1 M 8.5 pH Tris–HCl) and incubated for 10 min at 90 °C. The reduced pool of peptides was desalted by solid-phase extraction on reversed-phase SPE cartridges DSC-18 in the microcentrifugation column using a 100 μL 50% acetonitrile with 0.1% TFA solution for the elution. Eluted peptides were concentrated in a SpeedVac and resuspended in 15 μL 5% acetonitrile with 0.1% TFA, followed by mass-spectrometry analysis.

### 4.6. Peptide and Protein Identification

Tandem mass spectra were analyzed by PEAKS Studio version 8.0 software (Bioinformatics Solutions Inc., Waterloo, ON, Canada). The custom database was built from the UniProt database *T. aestivum* (UP000019116) combined with chloroplast and mitochondrial proteins (130,673 records). The database search was performed with the following parameters: a fragmentation mass tolerance of 0.05 Da; parent ion tolerance of 10 ppm; fixed modification—carbamidomethylation; variable modifications—oxidation (M) and acetylation (Protein N-term). The resulting protein list was filtered by a 1% false discovery rate (FDR). The parameter “Digestion Mode” was set to “unspecific” for extracellular peptide identification. For the mass spectrometry analysis, the peptide size was limited to a maximum of 65 amino acids, as defined by the settings in PEAKS Studio version 8.0.

PEAKS Q was used for iTRAQ quantification “https://www.bioinfor.com/quantification/ (accessed on 2 June 2024)”. Normalization was performed by averaging the abundance of all peptides. Median values were used for averaging. Given that iTRAQ quantification typically underestimates the degree of real fold changes between two samples, differential protein screening was performed using a fold change ratio ≥ 1.20 and significance ≥ 20 on the basis of iTRAQ studies [98,99,100]. The fold threshold for changed protein fold-change ratios was set at ±1.20, which covers a 95% quantification area based on the normal distribution of two samples in all biological replicates [98,101]. Protein significance analysis was performed in PEAKS 8.0 (Bioinformatics Solutions Inc., Waterloo, Canada). A two-tailed t-test was used for the calculation of significant differences in protein ratios (*p* < 0.05).

### 4.7. Statistical Analysis

Two-component principal component analysis (PCA) was performed using the *scikit-learn* Python package (version 1.6) based on standardized iTRAQ intensity values of each protein group across the samples. Standardization and dimensionality reduction were performed in the *scikit-learn* package with default parameters. The columns “Coverage (%)”, “Avg. Mass”, and “#Peptides” from the PEAKS result tables were used for analysis of quality control metrics of the proteomic datasets. Peptide lengths were calculated as the difference between the start and stop positions of each peptide. Data visualization was made in Python using the seaborn package (v. 0.11.1) [102].

### 4.8. Analysis of Transcriptomic Data

Publicly available transcriptomic data of root tissues from two *T. aestivum* cultivars—ZM366 and CM42 [41]—exposed to drought stress were analyzed (NCBI BioProject accession: PRJNA950485). Quality control of the raw data was performed using FastQC v. 0.12.1 [103]. Trimmomatic v. 0.39 [104] was used to clip adapters and nucleotides of low quality. Reference genome and genome annotation were accessed via Ensembl Plants [105], and IWGSC v.60 was accessed. Illumina short reads were mapped to the reference genome using HISAT2 v. 2.2.1 [106]. Transcripts were de novo assembled using Stringtie v2.2 [107] with the -e mode applied for quantification. Next, orfipy [108] was used to predict open reading frames (ORFs) from the AUG start codon and to further translate ORFs to sequences of length over 30 amino acid residues. The longest ORF per transcript was selected. Differential gene expression analysis was performed using the DESeq2 package [109]. Transcripts with logFC > 1 and adjusted *p*-value (FDR) < 0.05 were considered upregulated, with logFC < −1 and adjusted *p*-value < 0.05—down-regulated. Translated ORFs of length under 200 amino acids were considered small ORFs (smORFs). Finally, SSP Prediction Tool [68] was used to determine smORFs encoding precursors of known SSP families based on homology to SSPs from other plants.

### 4.9. Annotation Methods and Functional Enrichment

The gene ontology (GO) enrichment analysis was conducted using the ShinyGO 0.80 tool “https://bioinformatics.sdstate.edu/go80/ (accessed on 2 June 2024)”. Representative proteins from each protein group were used as input, and GO terms with a false discovery rate (FDR) ≤ 0.05 were considered significantly enriched. In addition, Kyoto Encyclopedia of Genes and Genomes (KEGG) pathway enrichment analysis was performed using the DAVID tool “https://davidbioinformatics.nih.gov/ (accessed on 10 June 2024)”. A significance threshold of *p* < 0.05 was applied to identify enriched pathways [62,110].

## 5. Conclusions

In this study, morphological changes, proteome, and peptidome alterations were determined in the roots and the leaves of the control and the drought-treated wild wheat seedlings to reveal proteomic responses to long-term drought stress in the two organs. Comparative proteomic analysis identified 497 and 157 DAP groups in leaves and roots, respectively. The upregulated DAP groups in leaves were primarily involved in stress responses, such as oxidative stress and heat response, whereas those in roots were associated with responses to water deprivation and sulfur compound metabolic processes. In leaves, the most strongly upregulated proteins belonged to well-known stress-responsive groups, including DHNs, RRM domain-containing proteins, nsLTPs, and water stress and hypersensitive response domain-containing proteins. Root proteome analysis revealed pronounced changes in proteins involved in stress adaptation and redox regulation under drought conditions. Upregulated proteins in roots included DHNs, ME, and LEA under drought stress. Notably, only a small subset of proteins was shared between the two organs, reflecting distinct tissue-specific drought adaptation strategies. Additional analysis of the extracellular root peptidome revealed 2294 native peptides, including members of small secreted peptide (SSP) families. In the peptidomes of stress-induced plants, we identified 16 SSPs as well as peptides derived from proteins involved in cell wall catabolism, intercellular signaling, and stress response. These peptides represent potential candidates as regulators of drought responses. Our results help us to understand adaptation mechanisms and develop new agricultural technologies to increase productivity.

## Data Availability

The datasets that we have presented in this study are available in public repositories. The mass spectrometry proteomics data have been deposited in the ProteomeXchange Consortium via the PRIDE partner repository with the dataset identifier PXD057751. Reviewer access details. Log in to the PRIDE website using the following details: Project accession: PXD057751. Token: uCyx7erSjr4r. Alternatively, the reviewer can access the dataset by logging in to the PRIDE website using the following account details: Username: reviewer_pxd057751@ebi.ac.uk. Password: mdojSHEC9vVI.

## Supplementary Materials

The following supporting information can be downloaded at https://www.mdpi.com/article/10.3390/plants14142168/s1, Figure S1: The GO term analysis of the extracellular peptides. A. Functional analysis of precursor proteins of secretome peptides in control. B. Functional analysis of precursor proteins of secretome peptide under drought stress. X-axis represents the number of proteins in each GO Term. Y-axis is GO Terms. Table S1: Differentially expressed leaf proteins under 4 days of drought stress. Table S2: Differentially expressed leaf proteins under 8 days of drought stress. Table S3: Common significantly regulated proteins expressed in leaves under 4- and 8-day drought stress. Table S4: Differentially expressed root proteins under 8 days of drought stress. Table S5: Common significantly regulated proteins expressed in both roots and leaves under 8 days of drought stress. Table S6: List of native extracellular root peptides under normal conditions. Table S7: List of native extracellular root peptides under drought stress. Table S8: List of predicted SSP families identified in root peptidomes. Table S9: List of predicted SSP families identified in CM42 transcriptome data. Table S10: List of predicted SSP families identified in ZM366 transcriptome data.

## Abbreviations

The following abbreviations are used in this manuscript:

ABA	Abscisic acid
CAPE	Cap-derived peptide
CAT	Catalase
DAPs	Differentially abundant proteins
DAPgroups	Differentially abundant protein groups
DHN	Dehydrin
DIR	Dirigent protein
iTRAQ	Isobaric tag for relative and absolute quantitation
IDA	Inflorescence deficient in abscission
JA	Jasmonic acid
LC/MS	Liquid chromatography–mass spectrometry
LEA	Late embryogenesis abundant
ME	Malic enzyme
NodGRP	Nodule-specific glycine-rich protein
nsLTP	Non-specific lipid-transfer protein
ORF	Open reading frame
PEG	Polyethylene glycol
POD	Peroxidase
PSK	Phytosulfokine
RuBisCO	Ribulose-1,5-bisphosphatecarboxylase/oxygenase
ROS	Reactive oxygen species
SA	Salicylic acid
SOD	Superoxide dismutase
SOT	Sulfotransferase
SSP	Small secreted peptide
SubIn	Subtilisin inhibitor
TCA	Tricarboxylic acid cycle
TCEP	Tris(2-carboxyethyl)phosphine
TFA	Trifluoroacetic acid
CAA	Chloroacetamide
