# Supplementary figures and images for: The Proteomic and Peptidomic Response of Wheat (Triticum aestivum L.) to Drought Stress
Source: Plants (Basel). 2025 Jul 14;14(14):2168. doi: 10.3390/plants14142168 (PMC12298440; doi:10.3390/plants14142168)

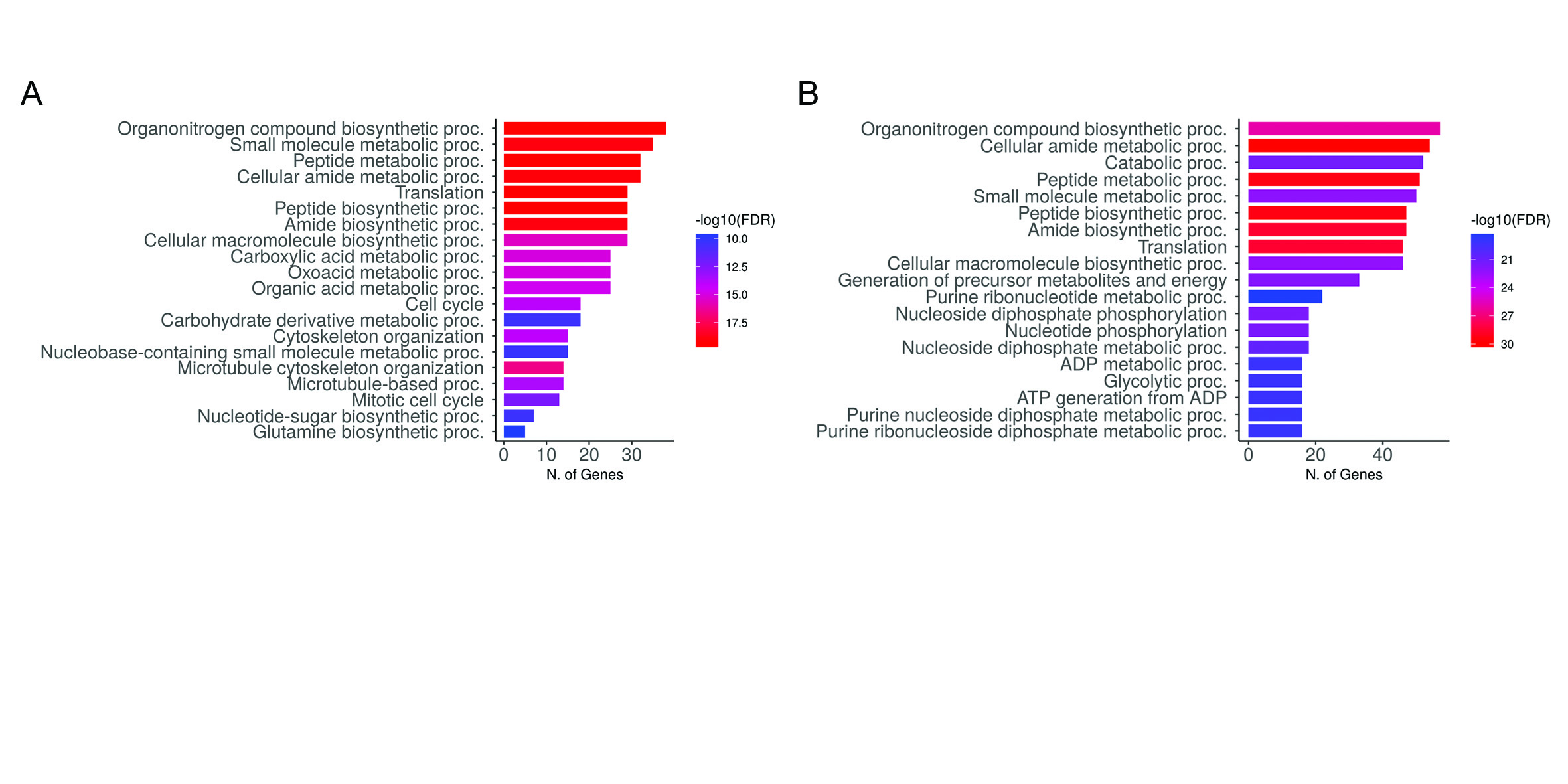

Supplement: Supplementary file 1 [file plants-14-02168-s001.zip › plants-3704949_Figure S1.jpg]
